# Cracking the nut of service-learning in nursing at a higher educational institution

**DOI:** 10.4102/curationis.v38i1.117

**Published:** 2015-03-20

**Authors:** Hester Julie, Oluyinka A. Adejumo, Jose M. Frantz

**Affiliations:** 1 School of Nursing, University of the Western Cape, South Africa

## Abstract

**Background:**

The readiness of academics to engage in the service-learning (SL) institutionalisation process is not accentuated in research on SL institutionalisation in South Africa. The argument has been advanced that SL scholarship and willingness of key stakeholders are crucial for SL institutionalisation at the academic programme level.

**Aim:**

The research focus of the study being reported here was on readiness of respondents to embed SL in the curricula of the nursing programme.

**Method:**

This study used a quantitative, exploratory and descriptive design. A self-administered structured questionnaire was used to collect data from a stratified sample comprising 34 respondents. The data were analysed for descriptive statistics using SPSS 19.

**Results:**

The demographic profile of the respondents indicated that 31 (66%) were between 31 and 50 years old; 36 (75.16%) had a minimum of 10 years’ nursing experience; 19 (39.6%) had a master's degree, two (4.2%) had a doctorate; and 29 (60.4%) had been employed by the school for a maximum of five years. The results indicated that the nurse educators were in need of SL capacity-building because 9 (18.8%) had limited or no knowledge of SL and 24 (50%) confused SL with other forms of community engagement activities. However, only 15 (33%) of the clinical supervisors and 13 (27%) of the lecturers indicated a willingness to participate in such a programme.

**Conclusion:**

The school was not ready to embed SL in the academic programme because of a lack of SL scholarship and willingness to remediate the identified theory–practice gaps.

## Introduction

### Problem statement

Although research on service-learning (SL) institutionalisation has proliferated both internationally and, lately, in South Africa, the researcher agrees with Bender (2007:138) in that that academics’ personal motivation tends to be downplayed in the institutionalisation process. The researcher argues that the success of SL institutionalisation is dependent on the readiness of key individuals at the delivery level of academic programmes to participate in the organisational change process implicit in SL institutionalisation. Therefore, investigating the SL status quo regarding personal-level engagement in disciplinary settings could provide a guide for focused intervention to improve the SL offering in the higher educational institution (HEI) community engagement environment (Bringle & Hatcher 2000:275).

### Aim

The research focus of the study being reported here was on readiness of respondents to embed SL in the curricula of the nursing programme.

### Background

Although national guidelines are available for higher education to institutionalise SL as a particular type of community engagement in South Africa, implementation of these guidelines has been inconsistent. Major concerns that were identified in working toward institutionalisation of SL in academic programmes included the non-operationalisation of SL in the institutional plans of HEIs according to Lazarus *et al*. (2008:58), pervasive conceptual confusion (Bender 2008:82) and a scarceness of conceptual frameworks to concretise community engagement activities (Hall 2010:24). The pivotal role that academics play in successful SL institutionalisation (Bender 2007; Bringle & Hatcher 1996; Erasmus 2007; Furco 2002b) is affirmed by the mandate given to HEIs. The South African SL policy documents, published by the Higher Education Quality Committee (HEQC), obliged HEIs to provide the necessary institutional support for academics to become involved in and develop SL (Bender *et al*. 2006:21–24; HEQC 2004:11). Regrettably, these documents do not give much attention to academics’ personal motivation in the institutionalisation process (Bender 2007:138). Hence, this article addresses the issue of readiness in terms of the status quo of SL scholarship and academics’ willingness to practise SL as prerequisites for SL institutionalisation. The latter symbolises the ‘nut’ referred to in the title, whilst ‘cracking’ refers to the intervention strategies that should be embedded in the change message of SL institutionalisation (Armenakis & Bedeian 1999:302).

Whilst the particular School of Nursing where the study was conducted was cognisant of the national policy imperative on SL as stipulated in the guidelines of the HEQC, operationalisation within the academic programmes had not been addressed. An intervention study was thus undertaken to develop an SL implementation framework for the School of Nursing using the multi-phased design and development model of Rothman and Thomas (1994). The factors that influenced the implementation of the HEQC's SL policy guidelines in the nursing programmes were explored during the first phase: problem analysis and project planning. During this phase, the research focused on the readiness of the school to institutionalise SL at both the organisational and individual level because SL scholars advocate a systems approach to SL institutionalisation (Furco 2002b:3; HEQC 2006:143). At the organisational level, the research question investigated whether the HEI had created an enabling environment for the school to institutionalise SL successfully in its academic programmes (Bender *et al*. 2006; Furco 2002b). The factors that were associated with readiness at organisational (school) level were those cited as critical success factors for SL institutionalisation (Furco 2002a:3), better known in South African terminology as SL ‘good practice indicators’ (Bender *et al*. 2006). Individual readiness was determined in terms of SL scholarship and willingness to participate in SL-capacitating activities (HEQC 2006:138).

This article reports on the findings related to the proportion of respondents who indicated previous exposure to SL, described SL accurately, requested SL information and who indicated a willingness to participate in SL capacity-building activities.

#### Definition of key concepts

**Community engagement** refers to the interactions and processes through which the expertise of the institution in the areas of teaching and research are applied in order to develop and sustain society (Osman & Petersen 2013:231).

**Service-learning (SL)** is conceptualised as an engaged pedagogy:

that integrates theory with relevant community service. Through assignments and class discussions, students reflect on their service in order to increase their understanding of module … content, gain a broader appreciation of a discipline, and enhance their sense of social responsibility. (HEQC 2006:32)

**SL institutionalisation** refers to the process through which SL is perceived and supported as being an essential component of the education process and thus embedded in the culture and organisation of the HEI. Tangible institutional commitment to mainstream SL is demonstrated by making SL an integral part of the infrastructure and everyday operations of the HEI's academic programmes, ensuring that SL is part of each student's academic experience (Fredericks, Holman & Canales 2002:3).

**SL scholarship** forms part of engaged scholarship in that all SL activities ‘reflect a knowledge-based approach to teaching, research, and service’ (McNall *et al*. 2009:319).

#### Contribution to field

The findings of the current study identified that the readiness of the implementers of an academic programme are an important factor to address during SL institutionalisation.

## Literature review

Since academics have been identified as the drivers for successful institutionalisation of SL (Bender 2007), a reasonable assumption is that one of the characteristics of an engaged HEI is that the institution would strategically build a critical mass of SL scholars across disciplines and faculties on campus (Furco 2002b). SL champions in higher education, regarded as change agents, are also advised to develop insight into both the individual and group change processes (Lamm & Gordon 2010:426; Whelan-Berry, Gordon & Hinings 2003) in order to ensure authentic commitment and to counter the natural tendency to resist change (Oreg 2003). Therefore, the discussion centres on the key questions proposed for the problem analysis process (Rothman & Thomas 1994:30).

### What is the nature of the discrepancy?

SL challenges the ‘dominant hegemonic practices of [*a*] disciplinary field’ (Osman & Petersen 2013:12). Therefore, any discrepancies in the implementation of SL at the School of Nursing could be benchmarked against the national standards outlined in the HEQC's SL policy guidelines (Bender *et al*. 2006:140–141; HEQC 2004:11). The anticipated outcome of this benchmarking exercise was *cracking the nut of SL* with the intention of moving the school toward authentic SL institutionalisation.

### Who should share responsibility for resolving the problem?

The HEQC (2004:5) mandates HEIs to integrate SL into their core business. It is therefore incumbent on HEIs to create an enabling environment by providing academics with an operationalised SL policy to facilitate the successful mainstreaming of SL modules into academic programmes (Saltmarsh, Hartley & Clayton 2009:1). Mainstreaming (SL institutionalisation) could be achieved by either developing a separate SL policy or infusing SL institutionalisation into all the relevant existing policies and institutional strategic plans (HEQC 2006:142).

**FIGURE 1:**
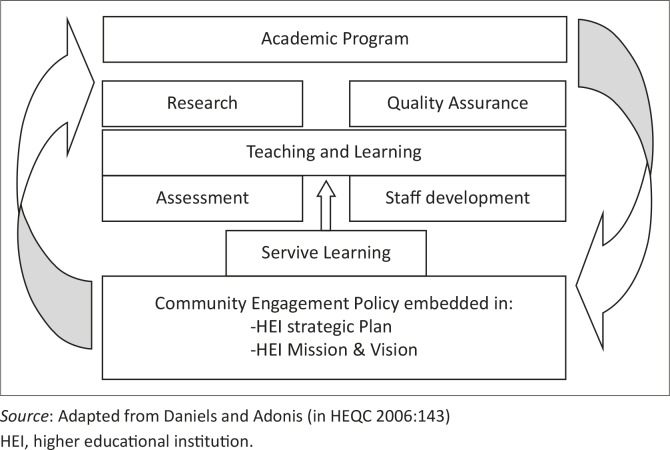
Infused service-learning institutionalisation framework.

At the base of the infused institutional framework is policy, which drives SL. The researcher, however, argues that the infusion of SL in these institutional structures related to the academic programme is essential but not sufficient for successful SL institutionalisation. The pivotal ingredient is the individual's readiness to engage with this SL process by committing to SL scholarship.

### Which conditions need to change to establish or support the required change?

The HEQC asserts that changes are required at different levels and that authentic support and involvement of institutional staff is critical (HEQC 2006:138). Hence, at the individual level, authentic SL institutionalisation requires awareness and a willingness to remediate possible theory–practice gaps. However, special attention should be paid to the assertion that ‘higher-order critical thinking outcomes [*are*] best embedded in a community of inquiry’ (Mthembu & Mtshali 2013:2). Therefore, at year- or nursing discipline-level, the expectation is that the communities of practice (the different teaching teams) would be willing to engage in a curriculum review process with the view of mapping modules most suited for SL pedagogy in the undergraduate nursing curriculum (Bender *et al*. 2006:18–19). The supposition is that shared assumptions and institutionalised experiences are foundational requirements for organisational change (Blackman & Henderson 2005:54). Consequently, the communities of practice can circumvent the negative effect of the potentially disparate SL practice theories of these individuals on the SL curriculum (Choi & Ruona 2011:62) and nurture institutional commitment (Julie, Daniels & Khanyile 2007).

## Research methods and design

### Design

The design was quantitative, exploratory and descriptive by nature. The design was quantitative because the researcher followed an objective and systematic process to obtain numerical data (Creswell 2009:3) so as to explore and quantify the problems pertaining to SL institutionalisation at individual level. The design was also descriptive in that the intention was to interpret accurately the characteristics of the group and the frequency with which certain phenomena occur (Polit & Beck 2008:752).

### Materials

#### Data collection instrument

A structured questionnaire consisting of both open- and closed-ended questions in English was developed with the assistance of a statistician. The questionnaire was structured into three sections.

*Section A* of the questionnaire focused on the socio-demographic information of the respondents. This section comprised six questions which requested information on the respondent's age group, gender, position and years employed at the School of Nursing, the highest nursing qualification and the total number of years’ nursing experience.

*Section B* was designed by the researcher, based on Bringle and Hatcher's work (2000:275). These authors identified the following indicators as evidence of academics’ involvement in SL institutionalisation: curriculum and module development, teaching team development activities and academics’ broad understanding of and support for SL and scholarship on SL (Bringle & Hatcher 2000:275). Hence, the nine yes/no items explored the SL scholarship needs of the respondents as determined by previous exposure to SL, request for SL information, willingness to participate in SL capacity-building and their understanding of SL.

*Section C* consisted of one open-ended question that asked respondents to describe their understanding of SL.

### Data collection method and procedure

The target population was informed electronically about the study and asked to participate. This was followed by face-to-face contact and respondents were requested to either complete the questionnaire electronically or submit a hard copy after informed consent had been obtained. The data were collected in two rounds because a number of new staff members were appointed at the beginning of 2011. The first round therefore took place during January to February 2011 and the second round during May to June 2011. This was done to ensure that new appointees had enough time to attend the induction and orientation programmes offered by both the university and the School of Nursing.

### Inclusion criteria

Academics (lecturers), clinical supervisors and senior academic officers had to be in the employ of the School of Nursing for at least four months prior to the research taking place in order to be eligible for inclusion in the study. The assumption was that, at that stage, they would have been fully oriented regarding the teaching and learning approaches of the undergraduate nursing programme and the teaching and learning policy of the university.

### Study population and sampling

Key stakeholders of the academic programme were involved to ensure cooperation, support and ownership (Rothman & Thomas 1994:29). Hence, the target population comprised all three employment categories – academics, clinical supervisors and senior academic officers – who were centrally involved with the undergraduate programme and employed by the school during the data collection period in 2011.

Stratified sampling was used because all the employment categories involved with the programme were included in the sample. Table 1 shows the sampling frame and sample size. The statistician used Cochran's (1977) formula to calculate the sample size.

**TABLE 1:** Sampling frame, required sample and actual sample size.

Target population	*n*	Required number	Actual number	% sample
Academics	25	16	22	45.9
Clinical supervisors	27	17	23	47.9
Senior academic officers	7	4	3	6.3
**Overall totals**	**59**	**37**	**48**	**100**

### Data analysis

All questions were analysed statistically using the Statistical Package for Social Sciences (SPSS), version 19.0 (IBM Corp., Armonk, NY 2010). The data were entered into a Microsoft® Excel spreadsheet and imported into SPSS for descriptive statistical analysis. The data are presented as frequencies and percentages in the form of tables and graphs.

### Context of study

The school is the largest residential nursing school in South Africa and has been offering the Bachelor of Nursing (B Nursing) degree as its core undergraduate programme since 2004 (Jeggels, Traut & Africa 2013:2). The school positions itself as an innovative School of Nursing and Midwifery in the country and advocates a community-, problem- and competency-based curriculum. The goal is to develop nursing practitioners who value and implement the primary healthcare approach and who are competent in metacognitive, problem-solving, partnership-building and self-directed learning skills (University of the Western Cape [UWC] 2013a).

## Results

This article reports data related to the demographics and the challenges to service-learning institutionalisation pertaining to the SL scholarship and capacity-building needs of the respondents.

### Demographic information

The demographic profile included gender, age group, employment category, highest nursing qualification, total number of years’ nursing experience and years employed at the current institution. See Table 2.

**TABLE 2:** Demographics of respondents.

Variable	Frequency	%
**Gender (*N*= 48)**		
Male	2	4.2
Female	46	95.8
Age group (years) (*N*= 47)*		
20–30	7	14.9
31–40	18	38.3
41–50	13	27.7
51–60	4	8.5
> 60	5	10.6
**Total years’ nursing experience (*N*= 48)**		
< 10	12	25.0
10–20	14	29.2
21–30	11	22.9
31–40	8	16.7
> 40	3	6.3
**Highest qualification (*N*= 48)**		
Diploma	10	20.8
Degree	11	22.9
Honours	6	12.5
Master's	19	39.6
Doctorate	2	4.2
**Years employed at institution (*N*= 48)**		
0–2	17	35.4
3–5	12	25.0
6–8	9	18.8
9–11	5	10.4
> 11	5	10.4
**Employment category (*N*= 48)**		
Lecturer	22	45.9
Clinical supervisor	23	47.9
Academic officer	3	6.3

*, One respondent did not provide details.

#### Age group

The findings indicate that the workforce of the school is mature in years because only 7 (14.9%) of the sample were 20–30 years old. The majority of respondents (*n* = 31; 66%) were between 31 and 50 years old, whilst 9 (19.1%) were older than 51 years.

#### Total years of nursing experience

It can be concluded that the School of Nursing had an experienced workforce because 36 (75.16%) of the respondents had a minimum of 10 years’ nursing experience compared to only 12 (25%) who had less than 10 years’ nursing experience.

#### Highest qualification

The findings indicate that 10 (20.8%) of the respondents had a diploma, which is lower than the prescribed minimum qualification to teach on the bachelor's nursing programme. The findings also indicate that nursing scholarship may be compromised because only 19 (39.6%) of the respondents held a master's degree, whilst 2 (4.2%) had a doctorate at the time of data collection.

#### Years employed at institution

The majority of participants (*n* = 29; 60.4%) had been employed in the school for a maximum of 5 years, with only 14 (29.2%) having worked at the School of Nursing for between 6 and 11 years. The findings hint at a high turnover rate of employees at the School of Nursing.

#### Employment category

The representation of lecturers (academics) and clinical supervisors was almost equal, with 22 (45.9%) lecturers and 23 (47.9%) clinical supervisors, respectively. Hence, the sample reflected the perspectives of both the theoretical and clinical components of the nursing programmes.

### Challenges to service-learning institutionalisation

Since institutional change is strongly associated with transformational shifts that occur within individuals, the transformational mindset regarding SL within the School of Nursing was determined by the following question: What is the proportion of respondents who identified a need for SL capacity-building as determined by personal commitment to develop SL scholarship? The SL scholarship needs of the respondents were determined by their previous exposure to SL, their request for foundational SL material and their willingness to participate in SL capacity-building. The assumption was that awareness of SL theory–practice gaps would be the catalyst that moves individuals to the commitment described by Bender (2007).

### Identification of service-learning capacity-building needs

The SL capacity-building needs were based on the level of SL scholarship at the time of the research within the school as assessed by previous SL exposure, understanding of SL and self-identified SL training needs (requesting information) of the respondents. Overall willingness to participate in capacity-building activities was also determined.

### Previous exposure to service-learning

Respondents were asked about attendance at SL training sessions, awareness of HEQC policy guidelines and assessment criteria and SL discussions in their communities of practice. Table 3 provides a summary of responses.

**TABLE 3:** Previous exposure to service-learning.

Type of service-learning exposed to previously	Responses					
	Yes		No		Total	
	Frequency	%	Frequency	%	Frequency	%
Attended SL training sessions	4	48	44	91.7	48	100
Aware of SL assessment criteria	9	18.8	39	81.3	48	100
SL discussions in communities of practice	3	6.3	45	93.8	48	100

SL, service-learning.

### Respondents’ understanding of service-learning at the time of the research

Respondents were asked to describe their understanding of SL. These responses were coded according to the following four categories: correct understanding of SL; confusing SL with other forms of community engagement activities; limited or no knowledge of SL; and no response. Four (8.3%) of the respondents had a correct understanding, 9 (18.8%) had limited or no knowledge of SL, 11 (22.9%) did not indicate any response and 24 (50%) confused SL with other forms of community-engagement activities.

### Self-identified service-learning training needs

Respondents were also asked to indicate whether they needed training in the philosophy, theoretical foundations and development of SL modules and information on the national SL policy guidelines. The responses are summarised in Table 4.

**TABLE 4:** Self-identified service-learning training needs.

Service-learning training needs	Responses					
	Yes		No		Total	
	Frequency	%	Frequency	%	Frequency	%
SL theory	22	45.8	26	54.2	48	100
SL philosophy	19	39.6	29	60.4	48	100
Developing SL modules	16	33.3	32	66.7	48	100

SL, service-learning.

### Willingness to participate in service-learning capacity development

Twenty-six (54.2%) of the respondents indicated their willingness to attend SL training sessions. Overall willingness, assessed by combining training needs and willingness to participate in SL capacity development, was indicated by 31 of the respondents (64.6%). The clinical supervisors were the most willing group, with 15 (33%) indicating willingness, followed by academics with 13 (27%) and academic officers with 2 (4%).

## Ethical considerations

### Informed consent

The respondents were required to give written consent before the research project commenced (Creswell 2009:89). In addition, all prospective respondents were given an information letter explaining the purpose, objectives and basis for inclusion in the study, as well as the expectations of the researcher, ethical considerations (especially the right to withdraw at any stage of the study) and contact details of the researcher and research supervisor. The signed consent forms were dated and co-signed by a witness (Denscombe 2010:67–69). The instruments and methods used during the research process were explained to the respondents prior to data collection.

### Privacy and confidentiality

This issue refers to the researcher's commitment not to violate the individuals’ privacy or to breach confidentiality by unwanted exposure of information. In order to prevent the researcher from linking them to the data entered on the survey, the respondents were not expected to write any personal details on the questionnaire. No personal details that might identify a respondent will be included in the findings or any subsequent research reports and publications (Denscombe 2010:64–65). All questionnaires were given an identification number for control purposes and only the researcher was aware of the origin of the data (Jooste 2010:278). The participants’ privacy was ensured in that the researcher used appropriate sampling techniques.

### The right to freedom of choice and withdrawal

None of the respondents were coerced to participate in the study – they were informed about the proposed study and that the choice to participate was voluntarily. In addition, they were informed about their right to withdraw from the study at any time without any penalty or being required to give a reason (Jooste 2010:279).

### Avoiding harm

Since the researcher was ethically bound to protect the respondents against any known physical and emotional harm, the information letter stated explicitly that no harm was associated with this study (Jooste 2010:280).

### Data protection

All the information collected and the communications related to the study were kept confidential because only those who were directly involved with the research had access to it. The instruments and the data will be kept in a safe place for at least five years after the results had been published, after which it would be destroyed in accordance with the institution's policy (Jooste 2010:278).

## Trustworthiness

### Reliability

Cronbach's alpha coefficient was used to test the internal consistency amongst scale items (Creswell 2009:149). The Cronbach's alpha coefficient yielded was 0.89, indicating high internal consistency.

### Validity

Validity refers to the degree to which an instrument measures what it is supposed to be measuring (Brink *et al*. 2005:159). The face and content validity of the questionnaire were determined, after which the instrument was pretested and modified based on feedback received from four academics, a statistician and the study mentor regarding clarity and conceptualisation.

## Discussion

### Outline of the results

The problems and challenges experienced by the academics and clinical supervisors during the implementation of the HEQC's SL guidelines in the nursing programme were investigated. The discussion here focuses on the main issues that were identified in the problem analysis and project-planning phase of the study (Rothman & Thomas 1994:11). During this phase, baseline data were collected in order to identify the readiness of the School of Nursing with regard to implementation of SL in the academic programme. Therefore, the major discussion points will focus on the readiness of the organisation and the respondents related to the implementation of the HEQC's SL policy guidelines at the School of Nursing.

#### Characteristics of the respondents

The majority of respondents had been employed by the school for a maximum of five years whilst a quarter had been employed between zero and two years (Table 1), indicating that the workforce was relatively inexperienced. This was a result of a high employment turnover rate at the School of Nursing as a result of the contract post policy of UWC (UWC 2013a:20). These two factors constitute threats to the nursing programme in terms of continuity and commitment to the programme as confirmed in the recent external review report of the School of Nursing (UWC 2013a:20). The extremely high workload is given as a reason why many contract staff terminated their employment at the School of Nursing (UWC 2013a:22). The resultant disruption of the academic programme implies that academics are less inclined to participate in the SL activities.

The willingness of staff to participate was compounded further by the pressure to improve their academic credentials as corrective measures for the low scholarly output in nursing (UWC 2013a:15). The findings in Table 1 also suggest that the academic qualification of all employment categories (academics, clinical supervisors and academic officers) could be construed as being a threat to the institutionalisation of SL at the School of Nursing for two reasons. At the time of the research, almost half of the total workforce of the school was studying part-time toward a doctorate or master's degree, attempting to achieve the strategic targets set for research (UWC 2009:3). This trend was confirmed by the dramatic increase in the doctoral enrolments: 8 in 2007, 15 in 2010 and 26 in 2012 (UWC 2013b:33–37). The staff component of the doctoral enrolments for 2013 and 2014 were 13 and 15, respectively, according to the October Postgraduate Supervision Report of the School of Nursing (UWC 2013b). The implication for the study was that this personal goal would be given priority (Self, Armenakis & Schraeder 2007) over the proposed change processes associated with SL institutionalisation at the school. The researcher, as the innovator of SL pedagogy at the School of Nursing (Julie, Daniels & Adonis 2005; Julie *et al*. 2007), therefore had to pay special attention to the individuals’ responses to the changes implicit in SL institutionalisation (Herold *et al*. 2008:349), regarded as the individual's readiness (Lamm & Gordon 2010:426).

#### Service-learning scholarship status at the School of Nursing

The primary focus of this article is the findings related to SL institutionalisation at the individual level because Bender (2007:138) submits that academics’ personal motivation is not foregrounded in the SL institutionalisation process. Since institutional change is strongly associated with transformational shifts that occur within individuals, their willingness to engage in SL scholarship activities was explored. The assumption was that awareness of SL theory–practice gaps (Armenakis & Bedeian 1999) would be catalytic in propelling individuals to participate in an SL intervention research project.

#### Previous service-learning exposure

The findings in Table 3 regarding previous exposure to SL clearly depict an SL theory-practice gap at the School of Nursing. The vast majority (*n* = 39; 81.3%) of participants were unfamiliar with the national SL policy guidelines, untrained in SL methodology and had no SL discourse in their respective communities of practice. This finding is a concern for various reasons. From 2003–2005, the School of Nursing participated in the national Community – Higher Education – Service Partnerships (CHESP) initiative in that the Gender-Based Violence SL module was developed as part of the Monitoring, Evaluation and Research Programme of CHESP. This contributed to the drafts of the HEQC's Policy Guidelines for community engagement and SL (Julie *et al*. 2005:3; 2007:52; Lazarus 2007:96). Another reason is that the CE Unit has been conducting SL development programmes since 2005 and three academics from nursing participated in the first SL capacity development programme aimed at developing SL modules (Adonis 2005:4). However, it should be noted that the educational landscape in nursing has changed drastically since 2006 because of a ministerial decision that UWC would be the only enrolling tertiary institution for undergraduate nursing in the Western Cape (UWC 2013a). The school thus had to contend with major internal and external situational crises, such as the increased student numbers and high staff turnover rates referred to under ‘Demographic profile’.

#### Personal commitment to service-learning scholarship

A surprise finding reflected in Table 4 was that only a third of the respondents requested theoretical information related to developing SL modules. This constituted a serious threat for institutionalising SL in the nursing programme, given that involvement in and support for SL by academics are cited as being two of the strongest indicators for successful institutionalisation (Bender 2007:134; Furco 2002a). The gravity of this situation was compounded by a lack of SL scholarship amongst the respondents as reflected in Table 3. These findings therefore confirm the need for change agents to determine the readiness of prospective collaborators before embarking on any of the organisational changes that were anticipated for the institutionalisation of SL at the School of Nursing (Herold *et al*. 2008). However, when the training needs and willingness to participate in SL capacity development were combined, the overall willingness was more promising. The findings also revealed that the majority of respondents were not conversant with the institutional SL policy statements reflected in the Institutional Operating Plans. The clinical supervisors were more willing than the academics to broaden their SL knowledge. These differences could be ascribed to the perception that SL is concerned with service delivery and therefore assumed to be the primary responsibility of the clinical supervisors.

The low proportion of academics indicating willingness is a concern if we concede that ‘curricular community engagement’ is a scholarly endeavour that ‘moves beyond the service experience’ (Bender 2007:128). Since the development of SL modules at the School of Nursing would rest primarily with the academics, it was necessary to turn around the negative dispositions towards SL. Organisation change researchers (Lamm & Gordon 2010:434; Wright & Pandey 2010:77) advocate psychological empowerment activities aimed at shaping ownership-taking behaviour for the change. This stance is reiterated by Bender (2007:138) for the institutionalisation of SL. The researcher thus had to use strategies that would ensure that the ‘buy-in’ from the individuals would be authentic (HEQC 2006:138) so as to counter the natural tendency to resist change (Oreg 2003).

#### Current understanding of service-learning at the School of Nursing

The findings related to the conceptual understanding of SL revealed that conceptual confusion was prevalent amongst both academics and clinical supervisors at the School of Nursing because only 8.3% provided a correct understanding of SL. Although this finding reflects a national trend, as stated by Bender (2008:81), it is imperative that the school develop an operational definition of SL so as to differentiate SL from other types of community-engagement curricular activities, because conceptual clarity is critical to SL institutionalisation (Bender 2007:137). These findings underscore the importance of Lazarus’ (2007:99) remark, ‘if higher education takes its reconstruction and development role seriously, its leaders will need to promote, support and reward a scholarship of CE’. Therefore, the larger intervention study attempted to create alignment between the institution's stated SL policy and the nursing programme requirements during the development of the SL implementation framework.

### Practical implications

The findings confirmed the readiness of academics in terms of SL scholarship as well as willingness, which is crucial to SL institutionalisation. The self-assessment tool for SL institutionalisation in higher education developed by Furco (2002b) should thus be broadened to include a dimension on the academics’ readiness.

## Recommendations

The management of the School of Nursing should address the existing conceptual confusion by developing an operational definition for SL in order to differentiate it from other forms of community engagement.

SL champions should be identified across the four year-levels of the programme and provide the necessary enabling environment in order to motivate academics to participate in SL capacity-building.

## Limitations of the study

This study was confined to one school that had a relatively high staff turnover. Hence, the study should be extended to other faculties in the university.

## Conclusion

This article reported on the individual's readiness as a crucial component for SL institutionalisation at the programme implementation level. In terms of ‘cracking the nut’, the results revealed that the respondents were not ready for SL institutionalisation because of a lack of SL scholarship and willingness to remediate the self-identified theory-practice gaps.
